# Co-Transplantation of GDNF-Overexpressing Neural Stem Cells and Fetal Dopaminergic Neurons Mitigates Motor Symptoms in a Rat Model of Parkinson’s Disease

**DOI:** 10.1371/journal.pone.0080880

**Published:** 2013-12-03

**Authors:** Xingli Deng, Yuanxin Liang, Hua Lu, Zhiyong Yang, Ru’en Liu, Jinkun Wang, Xiaobin Song, Jiang Long, Yu Li, Deqiang Lei, Zhongtang Feng

**Affiliations:** 1 Department of Neurosurgery, 1st Affiliated Hospital of Kunming Medical University, Kunming, Yunnan, China; 2 Cancer Center, Albert Einstein College of Medicine, New York, United States of America; 3 Department of Neurosurgery; China-Japan Friendship Hospital, Beijing, China; 4 Department of Neurosurgery, Union Hospital, Huazhong University of Science and Technology, Wuhan, Hubei, China; University of São Paulo, Brazil

## Abstract

Striatal transplantation of dopaminergic (DA) neurons or neural stem cells (NSCs) has been reported to improve the symptoms of Parkinson’s disease (PD), but the low rate of cell survival, differentiation, and integration in the host brain limits the therapeutic efficacy. We investigated the therapeutic effects of intracranial co-transplantation of mesencephalic NSCs stably overexpressing human glial-derived neurotrophic factor (GDNF-mNSCs) together with fetal DA neurons in the 6-OHDA rat model of PD. Striatal injection of mNSCs labeled by the contrast enhancer superparamagnetic iron oxide (SPIO) resulted in a hypointense signal in the striatum on T2-weighted magnetic resonance images that lasted for at least 8 weeks post-injection, confirming the long-term survival of injected stem cells in vivo. Co-transplantation of GDNF-mNSCs with fetal DA neurons significantly reduced apomorphine-induced rotation, a behavioral endophenotype of PD, compared to sham-treated controls, rats injected with mNSCs expressing empty vector (control mNSCs) plus fetal DA neurons, or rats injected separately with either control mNSCs, GDNF-mNSCs, or fetal DA neurons. In addition, survival and differentiation of mNSCs into DA neurons was significantly greater following co-transplantation of GDNF-mNSCs plus fetal DA neurons compared to the other treatment groups as indicated by the greater number of cell expressing both the mNSCs lineage tracer enhanced green fluorescent protein (eGFP) and the DA neuron marker tyrosine hydroxylase. The success of cell-based therapies for PD may be greatly improved by co-transplantation of fetal DA neurons with mNSCs genetically modified to overexpress trophic factors such as GDNF that support differentiation into DA cells and their survival in vivo.

## Introduction

Parkinson’s disease (PD) is one of the most prevalent diseases of aging. Although it has been recognized for many years that PD results from the degeneration of dopaminergic (DA) neurons in the nigrostriatal system [1], current therapies such as dopamine replacement and stereotaxic surgery yield only modest improvements in symptoms [2] and there is no treatment to halt disease progression.

Cell-based therapies for PD aim to replace lost nigral DA neurons with functional DA neurons derived from autologous or homologous neural stem cells (NSCs) or fetal DA neurons [3], and indeed both animal studies and clinical reports have provided evidence for the potential of NSCs (especially those derived from ventral mesencephalon) to differentiate into DA neurons in the adult mammalian brain [4], [5]. Thus, transplantation of NSCs [6] or fetal DA neurons [7]–[9] provide a potential therapeutic option for late-stage PD, and both striatal reinnervation and behavioral recovery have been observed in animal models and PD patients. However, the limited survival and poor function integration of transplanted cells remain major impediments to full clinical efficacy [10]–[14].

In an effort to improve the therapeutic effects of cell transplantation, we co-grafted mesencephalic neural stem cells (mNSCs) modified to express glial-derived neurotrophic factor (GDNF) together with fetal DA neurons into the 6-hydroxydopamine (6-OHDA) rat model of PD. This co-transplantation strategy reduced apomorphine-induced rotation, a Parkinsonian motor symptom in rats, improved NSCs survival in vivo, and promoted greater differentiation of NSCs into DA neurons compared to separate transplantation of NSCs or fetal DA neurons alone. This co-transplantation strategy may improve the clinical outcome of cell-based therapy for treatment of PD.

## Materials and Methods

### 1. Cell Culture

The human astrocytoma cell line U251 was purchased from the Cell Bank of the Chinese Academy of Sciences, Shanghai, China. Cells were cultured in Dulbecco’s modified Eagle’s medium (DMEM) (Life Technologies, USA) containing 10% heat-inactivated fetal bovine serum (FBS) (Life Technologies, Catalogue number [No.]: 12483020) at 37°C under a humidified 5% CO_2_ and 95% air atmosphere.

### 2. Animals

Fetal Sprague-Dawley (SD) rats at embryonic day 14 (E14) and E18 and adult male SD rats were obtained from Vital River Laboratory Animal Technology Co. Ltd. (Beijing, China). Adult males (body weight: 250±10 g; 5–6 weeks of age) were housed at 20−25°C and 50%±5% humidity with *ad libitum* access to food and water under a 12∶12 h light/dark cycle. The protocols in this study were approved by institutional review board and the Animal Care and Use Committee of Kunming Medical University (2009KMUL029).

### 3. Isolation and Culture of Ventral Mesencephalic Neural Stem Cells (mNSCs) and Fetal DA Neurons

Ventral mesencephalic neural tissue was obtained from fetal SD rats at E14 for isolation of mNSCs and at E18 for isolation of fetal DA neurons. Single cell suspensions were prepared as described in a previous report [15].

**Figure 1:**
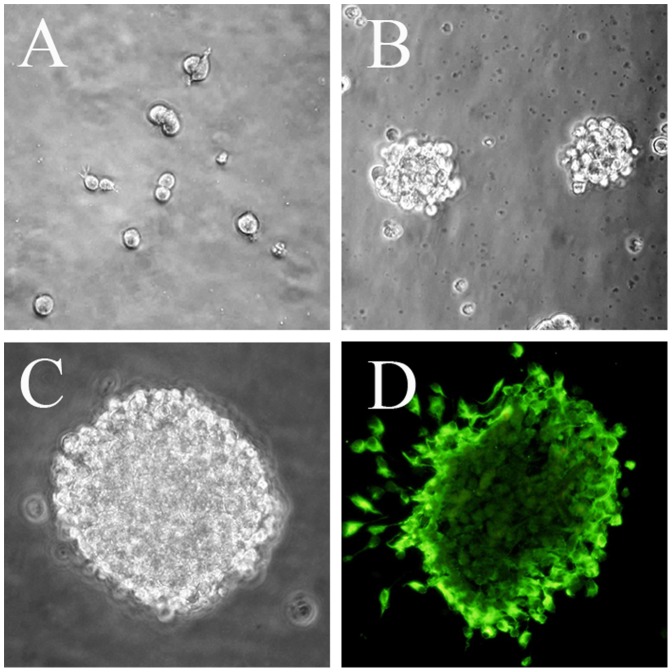
Identification of mNSCs in vitro. (A) Dumbbell-shaped mitotic cells were identified 72 hours after plating mesencephalic cells from E14 rats. (B) By 4 days in vitro (DIV), neurospheres had grown in size and detached from the culture substrate. (C) A floating 7 DIV neurosphere. (D) Fluorescence photomicrogragh showing a neurosphere at 7 DIV immunoreactive for the stem cell marker nestin. Scale bar = 100 µm. Green: FITC.

Mesencephalic NSCs (Figure 1) were cultured in serum-free NSC complete culture medium (a 1∶1 mixture of DMEM/Ham’s F-12 medium or DMEM/F12; Life Technologies, No. 11320-033) supplemented with (all from Life Technologies) 1% N2 (No. 17502048), 20 ng/ml recombinant human epidermal growth factor (EGF, No. PHG0311), 20 ng/ml recombinant human fibroblast growth factor (bFGF, No. PHG6015), and 2 mMol/L glutamine (No. 21051024). Half of the medium was replaced after 3−4 days and cells were passaged after 5−7 days. During passage, neurospheres were collected by centrifugation, mechanically dispersed into a single cell suspension by pipetting, and subcultured at 1×10^5^ cells/ml at 37°C under a 5% CO_2_ atmosphere.

**Figure 2:**
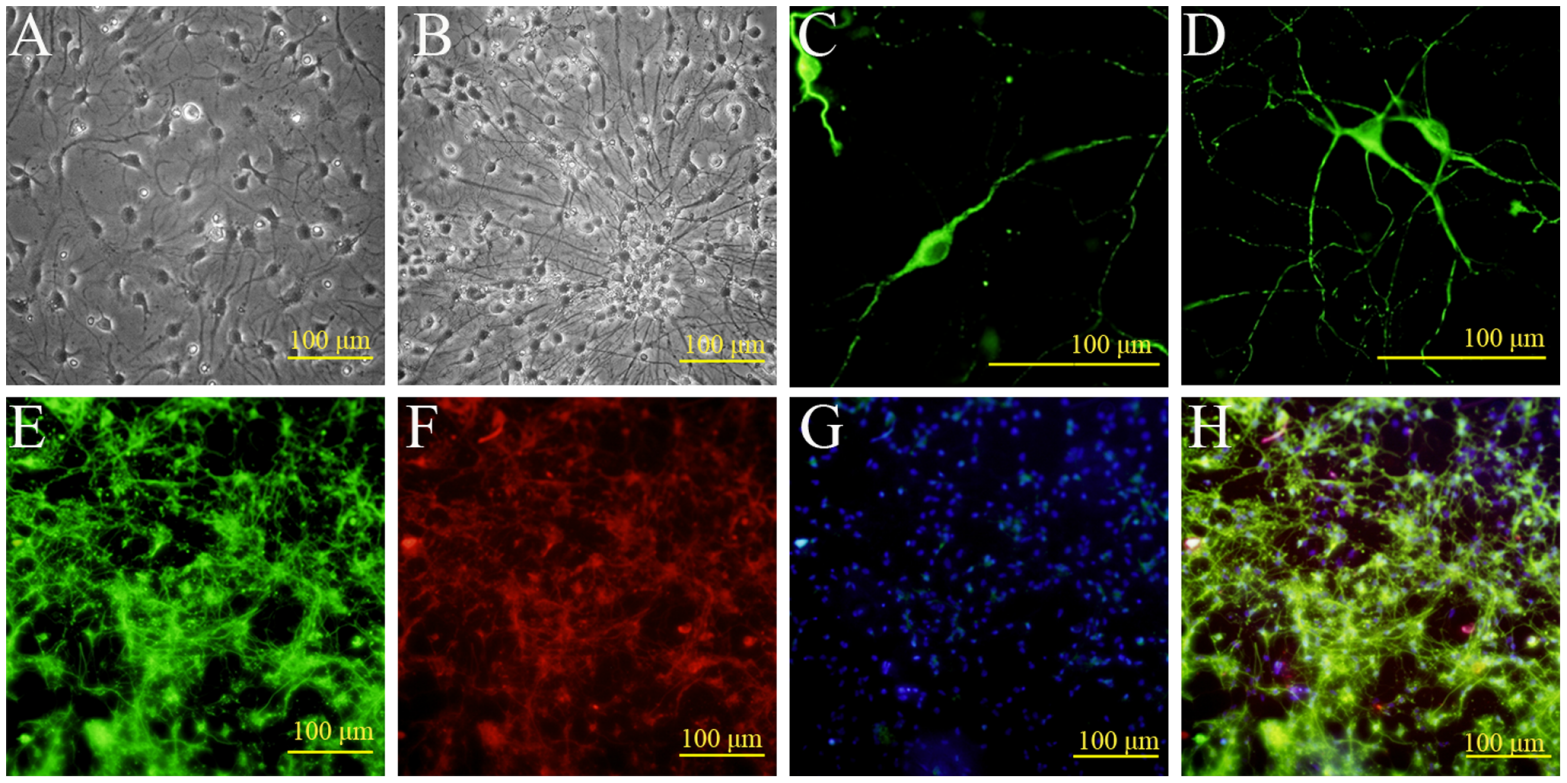
Identification of fetal DA neurons in vitro. Fetal DA neurons were isolated from E18 rat ventral midbrain. (A) Phase-contrast photomicrograph showing cells beginning to elaborate neural processes at 4 DIV. (B) With time in culture, neurites grew longer and thicker. (C, D) Immunofluorescence staining at 9 DIV showing TH-immunopositive neurons with a variety of distinct morphologies (unipolar, bipolar, and multipolar). Green: FITC. (E, F) Fluorescence photomicrograph showing that many cells express both the neuronal marker β-III-tubulin and the DA marker TH. Green: FITC; Red: TRITC. (G) DAPI (blue) nuclear counterstaining; (H) Merged image of E, F, and G. Scale bar = 100 µm.

Fetal DA neurons (Figure 2) were cultivated in neurobasal medium (Life Technologies, No. 21103049) supplemented with 1% fetal bovine serum, 1% B-27 (No. 17504044), and 0.001 mMol/L β-mercaptoethanol (No. 21985023) for 24 h, and then treated with 6.7 µg/ml fluorouracil (5-FU) (Sigma, USA, No. F6627-1G) for 24 h to suppress the growth of non-neuronal cells [16]. Thereafter, fetal DA neurons were maintained in neurobasal medium supplemented with fetal bovine serum, B-27, and β-mercaptoethanol.

### 4. Identification of Ventral mNSCs and Fetal DA Neurons in vitro by Immunostaining

#### 4.1. Ventral mNSCs

Neurospheres were collected by centrifugation, resuspended in DMEM/F12 plus 1% FBS (Gibco) and 1% N2, and seeded on polylysine-coated coverslips in culture plates. Cells were incubated at 37°C under 5% CO_2_ for 1 h to allow attachment and then stained with a fluorescein isothiocyanate (FITC)-labeled antibody against the stem cell marker nestin (Abcam) [17].

#### 4.2. Differentiation in vitro

Neurospheres were collected by centrifugation, resuspended in neural stem cell differentiation medium (DMEM/F12, 1% fetal bovine serum, 1% N2), seeded on polylysine-coated coverslips, and cultured at 37°C under 5% CO_2_ for 7 days. After 7 days in culture, cells were stained with antibodies against β-III-tubulin [18] and glial fibrillary acidic protein (GFAP) [19] (all from Abcam), immunocytochemical markers of neurons and astrocytes respectively.

#### 4.3. Fetal DA neurons

Primary cultured mesencephalic neurons were fixed in 4% paraformaldehyde, permeabilized with 0.25% Triton X-100, blocked in 3% BSA, and incubated for 12 h at 4°C with a rabbit anti-tyrosine hydroxylase (TH) antibody [20] and a mouse anti-β-III-tubulin antibody. Immunolabeling was visualized by staining with tetramethyl rhodamine isothiocyanate (TRITC)-conjugated goat anti-rabbit IgG andFITC-conjugated goat anti-mouse IgG (37°C for 1 h). Cell nuclei were counterstained with DAPI, and observed and photographed under a confocal microscope (OLYMPUS, Japan).

### 5. Superparamagnetic Iron Oxide (SPIO) and Enhanced Green Fluorescent Protein (eGFP) Double-Labeling of GDNF-Overexpressing mNSCs

The cDNA sequence of human *GDNF* (GenBank BC069369) was amplified from the human astrocytoma cell line U251 using forward 5′-CG*AAGCTT* **GCCACC** ATGAAGTTATGGGATGTCGTG-3′ (containing a *Hin* dIII site, a **Kozak** sequence, and an ATG start codon) and reverse 5′-GC*GGATCC*CAGATACATCCACACCTTTTAGC-3′ (containing a *Bam* HI site). The human *GDNF* PCR product was sequenced to verify accuracy, then digested with *Hin*dIII and *Bam*HI and ligated into the plasmid vector peGFPN1 (Clontech, Germany) containing a reporter gene encoding enhanced green fluorescent protein (eGFP) to yield the recombinant plasmid peGFPN1-GDNF. The sequence and restriction map of peGFPN1-GDNF were verified and the plasmid was then transfected into mNSCs using the Amaxa™ Basic Nucleofector Kit (Germany, VPI-1003) for primary mammalian neurons.

Stably transfected mNSCs (GDNF-mNSCs) were selected in medium containing G418 (Invitrogen). Cells from the third generation were labeled with SPIO (AMI-25, Advanced Magnetic) using the FuGENE HD transfection reagent (Roche, Swaziland). Prussian blue staining and transmission electron microscopy (HITACHI, Japan) were used to identify SPIO particles in transfected cells [21], [22].

### 6. RT-PCR

After GDNF-mNSCs were selected in medium containing G418, total RNA was isolated using the RNeasy Plus Mini Kit (QIAGEN, 74134) and purity determined by absorbance at 260 and 280 nm (A260/280). The integrity of the RNA was verified by formaldehyde denaturing gel electrophoresis. cDNAs were synthesized using the Revert Aid™ First Strand cDNA Synthesis Kit (MBI Fermentas, K1611) according to the manufacturer’s instructions. The cDNAs were amplified using the primers 5′-AGTTATGGGATGTCGTGG-3′ (forward) and 5′-GAAGCACTGCCATTTGTT-3′ (reverse) to determine GDNF mRNA expression (product length: 255 bp). Expression was normalized to that of β-actin (amplified using forward 5′-GGACATCCGTAAAGACCTG-3′ and reverse 5′-AAAGGGTGTAACGCAACTAA-3′, product length: 300 bp). All transcripts were amplified by initial denaturation at 94°C for 3 min, 25 cycles of 94°C for 45 s, 50°C for 1 min, and 72°C for 1 min, and a final extension at 72°C for 10 min. The PCR products were subjected to 1% agarose gel electrophoresis and the abundance of each mRNA was normalized to β-actin using ImageJ software (NIH, USA).

### 7. Immunofluorescence

For immunocytochemistry staining, mNSCs were cultured for 1 hour in DMEM/F-12 lacking bFGF and EGF, but containing 1% bovine serum. The cells were washed three times in PBS, fixed in 4% paraformaldehyde for 10 min at room temperature (RT), and permeabilized with PBS plus 0.25% Triton X-100 (PBST) for 10 min at RT (or 15 min at RT if the target antigen was a nucleoprotein). After three washes in PBS, the cells were blocked with 1% BSA for 30 min at RT and incubated with anti-nestin (mouse monoclonal, 1∶1000, Abcam, No. ab6142) and anti-GDNF (rabbit polyclonal, 1∶20, No. ab18956) overnight at 4°C. Immunolabeling was visualized by incubation (1 h at RT) with rhodamine-conjugated Affinipure goat anti-mouse or anti-rabbit IgG (1∶100, Zymed, USA). After three washes in PBS and two in deionized water, immunolabeled cells were counterstained with 50 µl DAPI at 37°C for 10 min and washed in sequence with methanol and ethanol. Cells were observed under a fluorescence microscope (OLYMPUS, Japan).

### 8. Western Blotting

Cells or tissues were lysed in Neuronal Protein Extraction Reagent (N-PER, Pierce, USA) on ice for 20 min and centrifuged at 13000 rpm for 5 min at 4°C. The supermatant protein concentrations were measured by the Bradford assay (Pierce, USA). Equal amounts of protein per gel lane were separated by 15% SDS-polyacrylamide gel electrophoresis and transferred to nitrocellulose membranes (Whatman, Germany). Membranes were blocked with 5% dry milk/Tris-buffered saline (TBS) for 1 h and then incubated overnight at 4°C with a GDNF antibody (rabbit polyclonal, 1∶50, Abcam, No. ab18956) and β-actin antibody (Abcam) in 5% dry milk/TBS. Following three washes with 0.1% Tween 20/TBS (TBST), blots were incubated with horseradish peroxidase (HRP)-conjugated anti-rabbit IgG (1∶5000, Abcam) at room temperature for 1 h and developed using a chemiluminescence system (KPL, USA). The relative protein expression was normalized to β-actin using Image J software.

### 9. Rat PD Model and Cell Transplantation

Seventy adult male SD rats were anesthetized with 50 mg/kg pentobarbital sodium by intraperitoneal (i.p.) injection and immobilized in a stereotaxic frame. 6-OHDA (12 µg/4 µl ascorbate-saline, Sigma, USA) was injected into the right medial forebrain bundle (MFB) (anteroposterior [A/P] = –3.6 mm from bregma; lateral [L] = +2.0 mm; ventral [V] = –7.7 mm from dura) and ventral tegmental area (VTA) (A/P = −6.0 mm; L = +0.5 mm; V = −8.0 mm) [7]. One week after surgery, apomorphine (APO, 0.25 mg/kg, Sigma,) was injected (i.p.) to induce rotational behavior, a behavioral endophenotype of this PD model. Beginning 10 minutes after APO administration, the absolute number of rotations over the next 30 min was counted, and the average rotations per minute (RPM) were calculated. The test was performed after experimental treatments (Table 1) as a measure of the severity of PD-like motor symptoms. Rats with an average RPM of less than six in the first 30 min after apomorphine administration were excluded from the transplantation experiments [23]. Thirty-six PD model rats were used for subsequent transplantation experiments.

**Table 1:** Mean rotation index.

Group	Rotation index (postgrafting RPM/pregrafting RPM)			
	2 w	4 w	6 w	8 w
Sham-operation	1.098±0.013#	1.163±0.015#	1.167±0.023#	1.272±0.032#
DA	0.533±0.016* #	0.530±0.014* #	0.573±0.012* #	0.613±0.016* #
control mNSCs	0.895±0.014* #	0.887±0.012* #	0.838±0.013* #	0.795±0.010* #
GDNF-mNSCs	0.850±0.009* #	0.808±0.012* #	0.755±0.019* #	0.695±0.014* #
control mNSCs+DA	0.458±0.015* #	0.438±0.020* #	0.383±0.020* #	0.357±0.015* #
GDNF-mNSCs+DA	0.410±0.017*	0.377±0.018*	0.237±0.014*	0.242±0.039*

*Note:* Values are means ± SD of 6 rats from each group.

* *P*<0.01 *vs.* Sham-operated group,

# *P*<0.01 *vs.* GDNF-mNSCs+DA group. peGFPN1-GDNF-transfected mNSCs (termed GDNF-mNSCs); mNSCs expressing empty vector (termed control mNSCs).

Approximately 2.5×10^5^ cells in 10 µl were injected into the ipsilateral striatum (right side) at A/P = +0.5 mm; L = +3.0 mm; V = −5.0 mm using a Hamilton syringe. Sham-treated rats (n = 6) were punctured at the same coordinates with the empty syringe. All other treatments were identical. Experimental animals were injected with fetal DA neurons, mNSCs expressing empty vector (control mNSCs), GDNF-mNSCs, control mNSCs+fetal DA neurons, and GDNF-mNSCs+fetal DA neurons (each n = 6). Apomorphine-induced rotational behavior was examined 2, 4, 6 and 8 weeks after cell transplantation. The ratio of the APO-evoked rotation rate after transplantation to the rotation rate before transplantation was used as a measure of improved motor function [24].

### 10. Survival, Migration, and Differentiation of Transplanted Cells

Survival and migration of transplanted stem cells were determined by MRI using intracellular SPIO as a contrast agent. All animals received an MRI scan 1, 2, 4 and 8 weeks after cell transplantation. Animals were anesthetized with 2% sodium pentobarbital (i.p.), mounted onto the stereotaxic frame to hold the animal, and MRI scans acquired using a Philips Intera 1.5 T MRI system (Netherlands) with a 5-inch surface coil. A three planar positioning scan was followed by gradient-echo T2-weighted (T2W/FFE) imaging using the following acquisition parameters: Repetition Time (TR) 388 ms, Echo Time (TE) 23 ms, flip angle 18°, field of view (FOV) 120 mm, matrix 205×256, slice thickness 2.0 mm, and scan time 2 min 8 s.

Migration and differentiation of transplanted cells were examined by immunofluorescence staining of brain sections. At 8 weeks after cell transplantation, all rats were heavily anesthetized by pentobarbital sodium (100 mg/kg, i.p.), and perfused transcardially with 100 ml PBS and 300 ml 4% paraformaldehyde in PBS (pH 7.4). Striatal tissues near the injection site were resected and immersed in 4% paraformaldehyde for an additional 12 h. Tissue blocks were embedded in OTC compound, frozen, and cut into 10-µm thick transverse slices using a Leica CM1950 cryostat (Germany). Frozen sections were incubated for 8 h at 4°C with anti-nestin (mouse monoclonal, 1∶1000, Abcam, No. ab6142), anti-TH (mouse monoclonal, 1∶500, Abcam, No. ab6211), anti-β-III-Tubulin (mouse monoclonal, 1∶1000, Abcam, No. ab14545), and anti-GFAP (rabbit polyclonal, 1∶750, Abcam, No. ab7779), washed in PBS, and then incubated with anti-eGFP (goat polyclonal, 1∶1000, Abcam, No. ab111258) for 8 h at 4°C. After washing in PBS, immunolabeling was visualized by incubation for 2 h at RT by FITC- or TRITC-labeled IgG (1∶100, ZSGB-BIO). Sections were observed under a confocal laser scanning microscope (OLYMPUS, Japan). Cells expressing eGFP (eGFP+), nestin (nestin+), tyrosine hydroxylase (TH+), glial fibrillary acidic protein (GFAP+), both nestin and eGFP (nestin+/eGFP+), both TH and eGFP (TH+/eGFP+), or both GFAP and eGFP (GFAP+/eGFP+) were counted in 5 sections from each animal and expressed relative to the total number of cells in the section. The rate of differentiation was expressed as the number of cells expressing both a neuronal marker and eGFP relative to the total number of eGFP-positive cells×100%.

### 11. Statistical Analyses

The SPSS12.0 statistical software package was used for statistical analyses. Results are presented as means ± standard deviation (SD). Statistical significance was evaluated by *t*-test or one-way analysis of variance (ANOVA) with least significant difference (LSD) test for post hoc analysis. A *P*<0.05 was considered statistically significant.

## Results

### 1. Identification of SPIO/eGFP Double-labeled GDNF-mNSCs and Fetal DA Neurons in vitro

Mesencephalic neural stem cells (mNSCs) were isolated from E14 rats as described (Figure 1). Three days after plating, most cells were solitary or in small aggregates of a few cells, but many exhibited morphological signs of mitosis (Figure 1A). After 4 days in vitro (DIV), these proliferating cells formed larger spherical colonies (neurospheres) that grew in size over the next 3−5 days and eventually detached from the substrate (Figure 1B, 1C). These neurospheres were strongly immunopositive for the stem cell marker nestin (Figure 1D). Fetal dopaminergic (DA) neurons were isolated from the ventral midbrain of E18 rats and identified in culture by the elaboration of neurites (Figure 2B−D) and immunoreactivity for the DA synthesis enzyme tyrosine hydroxylase (Figure 2C, 2D) and the mature neuron marker β-III-tubulin (Figure 2F).

**Figure 3:**
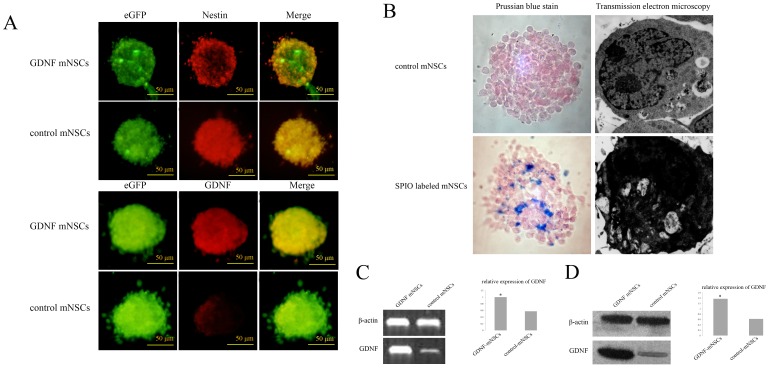
Identification of mesencephalic neural stem cells overexpressing GDNF (GDNF-mNCSs). (A) Fluorescence photomicrographs show that all neurospheres were immunoreactive for the NSCs marker nestin. The neurospheres of peGFPN1-GDNF-transfected mNSCs (termed GDNF-mNSCs) were strong immunopositive for GDNF compare to mNSCs transfected with peGFPN1. Green: FITC, Red: TRITC. Scale bar = 50 µm. (B) Prussian blue staining and transmission electron microscopy confirmed SPIO particles in the cytoplasm of mNSCs. (C) GDNF mRNA expression in GDNF-mNSCs compared to mNSCs transfected with peGFPN1 (termed control mNSCs) was determined by RT-PCR. β-actin was used as an internal control. (D) GDNF protein expression in GDNF-mNSCs and control mNSCs was determined by Western blot. Relative protein expression was normalized to β-actin. The data represents the mean ± SD. *P<0.05 *vs.* control mNSCs. peGFPN1-GDNF-transfected mNSCs (termed GDNF-mNSCs); mNSCs expressing empty vector (termed control mNSCs).

Neurospheres from cells stably transfected with the peGFPN1-GDNF plasmid demonstrated much higher GDNF protein expression than mNSCs transfected with the empty peGFPN1 plasmid (Figure 3A). These GDNF-mNSCs also expressing eGFP were then labeled with SPIO for in vivo detection by MRI. Prussian blue staining and transmission electron microscopy confirmed SPIO particles in the cytoplasm of mNSCs (Figure 3B). Furthermore, RT-PCR indicated enhanced GDNF mRNA expression in GDNF-mNSCs (about 2.1-fold higher than that in mNSCs transfected with the empty peGFPN1 vector) (Figure 3C), while Western blotting revealed approximately 2.2-fold higher GDNF expression (Figure 3D).

### 2. In vitro Differentiation of SPIO, eGFP Double-labeled GDNF-mNSCs

**Figure 4:**
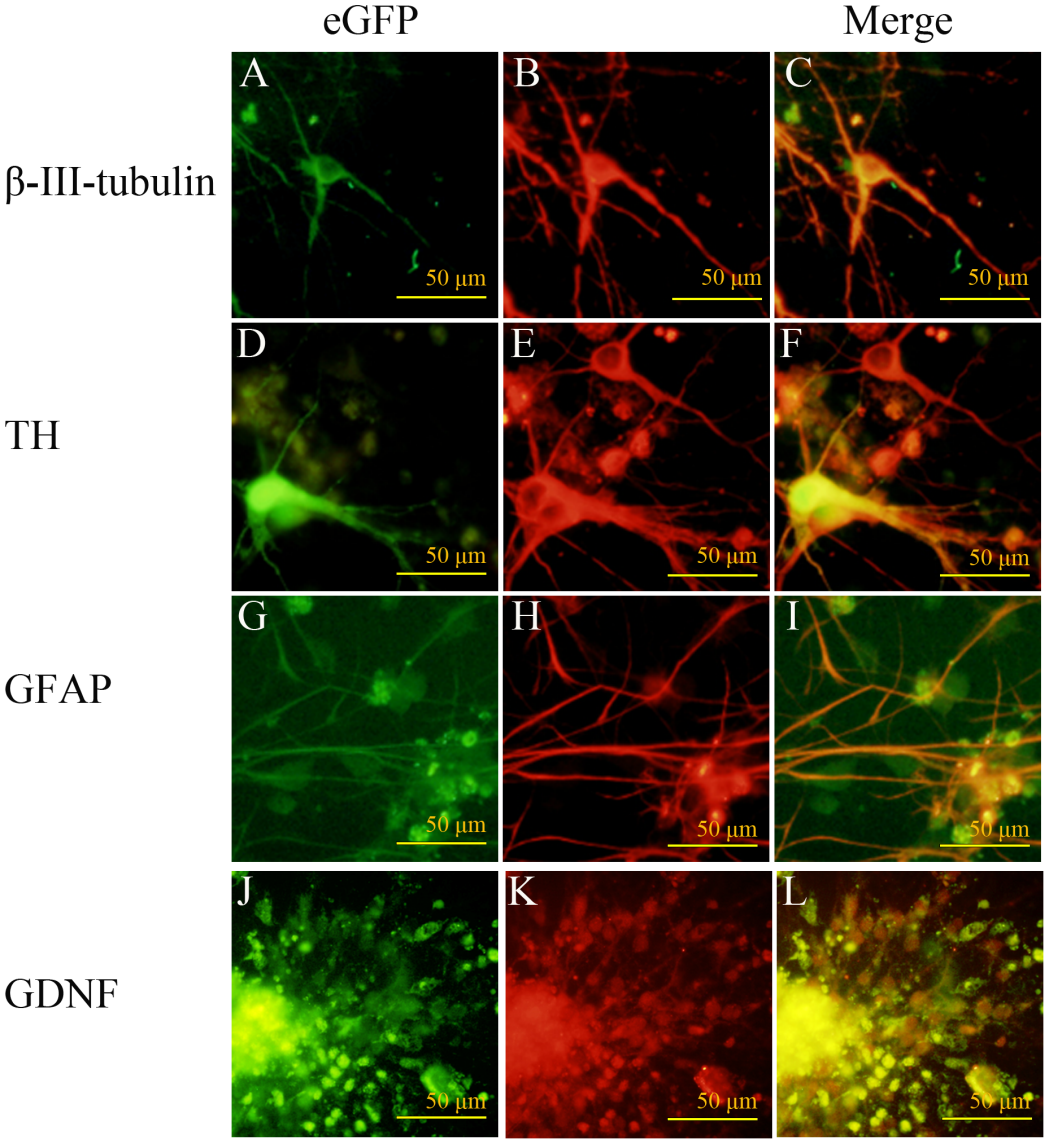
In vitro differentiation of SPIO, eGFP double-labeled GDNF-mNSCs. Fluorescence photomicrographs showing SPIO, eGFP double-labeled GDNF-NSCs growing in a differentiation medium for seven days. eGFP-positive cells (A, D, G, J), immunoreactive for the neuronal markers β-III-tubulin (B) and TH (E), the astrocyte marker GFAP (H), and GDNF (K). Images are merged in the last column (C, F, I, L). Scale bar = 100 µm. Green: FITC, Red: TRITC.

Neurospheres derived from SPIO, eGFP double-labeled GDNF-mNSCs were grown for seven days in a NSCs differentiation medium and cell phenotype examined by immunofluorescence staining (Figure 4). Cells were immunopositive for the neuronal marker β-III-tubulin (Figure 4B), the DA neuron marker TH (Figure 4E), or the astrocytic marker GFAP (Figure 4H). Moreover, cells still expressed GDNF (Figure 4K). Thus, SPIO, eGFP double-labeled GDNF-mNSCs are multipotent in vitro and continue to express GDNF.

### 3. Success Rate of the PD Model

Rats were injected with the dopaminergic neurotoxin 6-OHDA as described and then examined for a PD-like behavioral phenotype using the apomorphine (APO)-induced rotation assay. Of the 70 rats injected with 6-OHDA, 39 exhibited a minimum of 6 rotations/min over the first 30 min post-injection (55.7%), the criterion for successful PD-model induction.

### 4. Reduction of APO-induced Rotational Behavior after Cell Transplantation

Parkinson’s disease model rats (n = 36) were divided into 6 equal groups. One group was sham-treated while the other 5 groups were injected with fetal DA neurons (isolate and identified as in Figure 2), control mNSCs, GDNF-mNSCs, control mNSCs plus fetal DA neurons, or GDNF-mNSCs plus fetal DA neurons. All groups that received cell transplantation demonstrated a reduction in APO-induced rotation behavior as expressed by the after/before transplant rotational ratio compared to the sham-treated group (Table 1). The largest decrease in rotational ratio compared to the sham-operation group (1.272±0.032) was observed in the GDNF-mNSCs+DA group (0.242±0.039), (*P*<0.01) (Table 1). Thus, transplantation of multipotent stem cells or fetal DA neurons improved a behavioral endophenotype of PD, but transplantation of both fetal DA neurons and GDNF-overexpressing mNSCs resulted in the largest degree of improvement.

### 5. Survival, Migration, and Differentiation of Transplanted Cells

**Figure 5:**
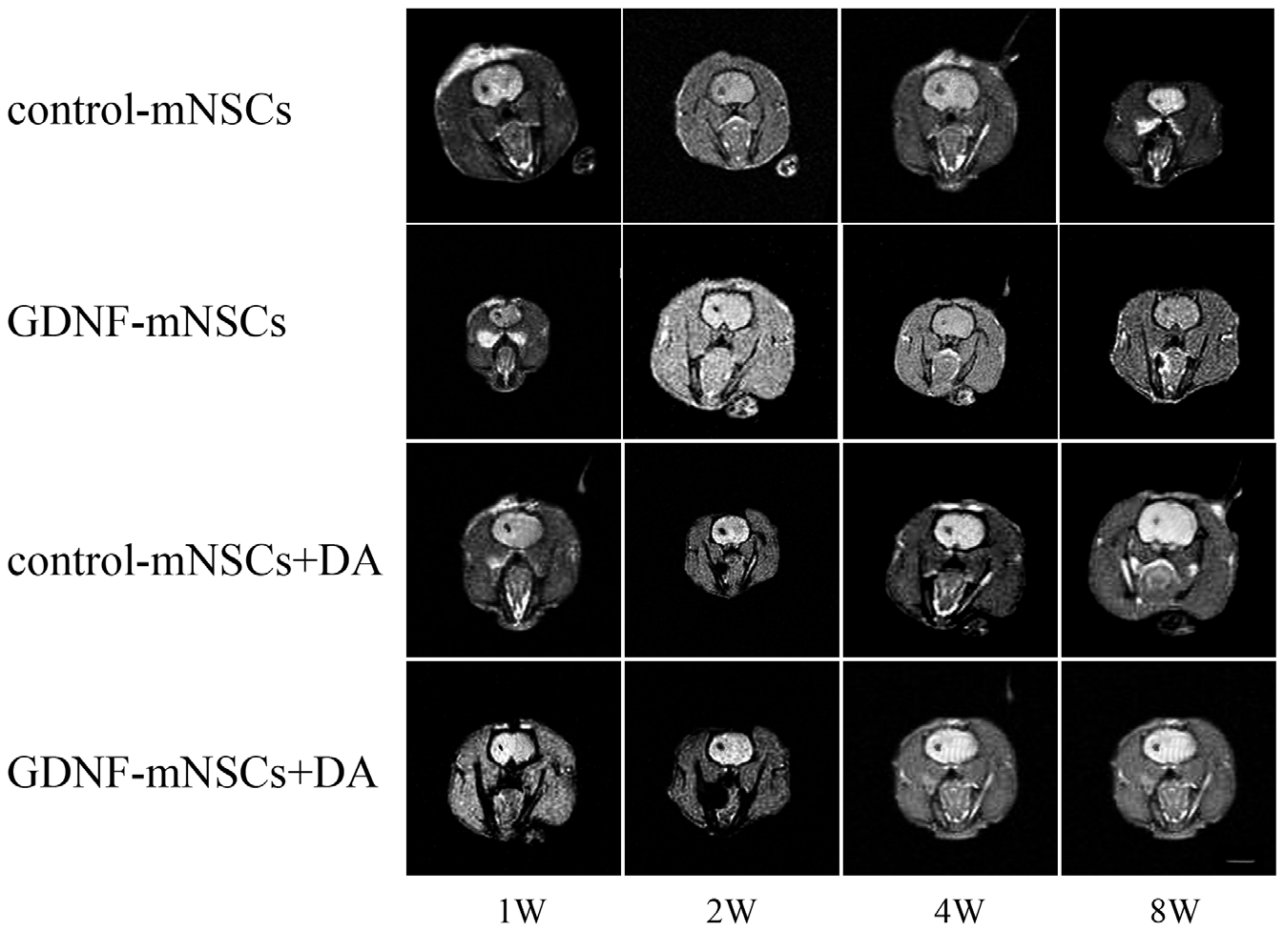
MR images (T2W/FFE) showing the survival of SPIO-labeled mNSCs in the striatum. Note the high density of SPIO-labeled mNSCs in each group at the site of injection (striatum), but no detectable extrastriatal signal.

**Figure 6:**
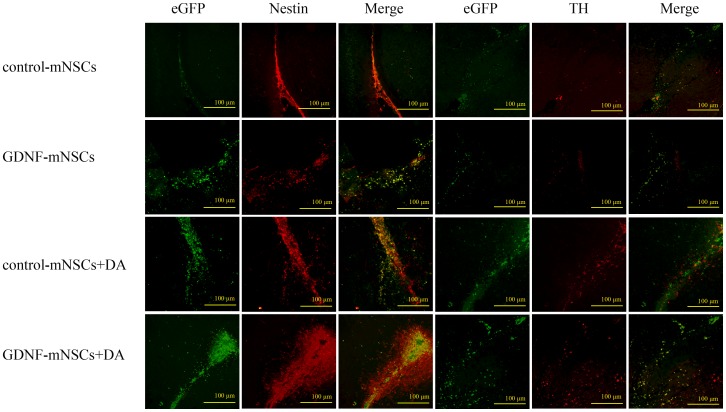
Survival and differentiation of transplanted cells in the striatum. Representative photomicrograph illustrating that many transplanted cells survived in the striatum. There were more eGFP^+^ (green), nestin^+^ (red), nestin^+^/eGFP^+^(merged yellow), TH^+^ (red), and TH^+^/eGFP^+^ cells (merged yellow) in the GDNF-mNSCs+DA group than in the other groups. Scale bar = 100 µm. Green: FITC, Red: TRITC.

Magnetic resonance images of anesthetized rats (Figure 5) and immunohistochemical staining of brain sections through the striatum (Figure 6) showed that almost all the injected cells were retained at the transplantation site. Injected stem cells survived in vivo as indicated by the hypointense signal in the striatum on T2-weighed MRI from cells labeled with superparamagnetic iron oxide (SPIO) at up to 8 weeks post-injection. Mesencephalic NSCs transfected with an empty vector remained largely undifferentiated as indicated by nestin staining, with only a few differentiating into neurons or glial cells (Figures 6). Survival of grafted cells was significantly higher in the GDNF-mNSCs+DA group than in all other groups as indicated by the total number of eGFP^+^ cells (all *P*<0.05). Moreover, the GDNF-mNSCs+DA group also exhibited the highest number of undifferentiated cells (919±66 Nestin+ cells), transplanted undifferentiated cells (236±19 Nestin+/eGFP+ cells), surviving DA neurons (185±16 TH+ cells), and transplanted mNSCs-derived DA neurons (86±12 TH+/eGFP+ cells,), but the lowest number of mNSCs-derived astrocytes (200±9 GFAP+ cells) (Table 2). Indeed, the rate of mNSCs differentiation into DA neurons was markedly higher in the GDNF-mNSCs+DA group than in rats injected with control mNSCs (7.3%±0.8%) or control mNSCs plus DA neurons (13.2% ±1.0%). While GDNF-mNSCs also demonstrated a high differentiation rate when injected alone (18.3%±1.9%), the total number of mNSCs-derived DA neurons was far lower than in the GDNF-mNSCs+DA group (86±12 vs. 20±3 TH^+^/eGFP^+^ cells) due to the smaller population of viable injected mNSCs (381±15 vs. 108±7 eGFP+ cells). Moreover, while co-injection of fetal DA neurons and control mNSCs also resulted in a relatively large number of surviving stem cells (eGFP+ cells), the lower rate of differentiation (13.2% ±1.0%) resulted in many fewer mNSCs-derived DA neurons. Thus, overexpression of GDNF and the presence of fetal DA neurons act synergistically to enhance stem cell survival and differentiation into DA neurons.

**Table 2:** Number of cells with specific expression phenotypes and DA neuron differentiation rate after cell transplantation into the rat striatum.

Group	Cell number						Differentiation rate			
	eGFP^+^	Nestin^+^	eGFP^+^/Nestin^+^	TH^+^	eGFP^+^/TH^+^	GFAP^+^	eGFP^+^/GFAP^+^	Nestin^+^ (%)	TH^+^ (%)	GFAP^+^ (%)
control mNSCs	65±11#	105±17#	37±6#	6±2#	5±1#	647±39#	36±4#	57.1±2.4#	7.3±0.8#	56.1±7.2#
GDNF-mNSCs	108±7#	494±11#	41±4#	26±4#	20±3#	324±15#	56±7#	38.1±1.6#	18.3±1.9#	52.6±5.4#
control mNSCs+DA	277±13#	592±10#	185±7#	112±8#	37±4#	344±18#	97±9#	67.1±3.7#	13.2±1.0#	35.3±3.9
GDNF-mNSCs+DA	381±15	919±66	236±19	185±16	86±12	200±9	115±5	85.7±5.1	22.5±3.0	30.1±0.33

There were more eGFP^+^, Nestin^+^, eGFP^+^/Nestin^+^, TH^+^, and eGFP^+^/TH^+^ cells and fewer GFAP^+^ cells in the GDNF-mNSCs+DA group than the other groups. (Differentiation rate = Number of cells expressing both a neuronal marker and eGFP/Number of eGFP-positive cells×100%). Values are means ± SD of 6 rats from each group.

# *P*<0.01 *vs.* GDNF-mNSCs+DA group. peGFPN1-GDNF transfected mNSCs (termed GDNF-mNSCs); mNSCs expressing empty vector (termed control mNSCs).

## Discussion

Although numerous studies have shown that transplantation of fetal DA neurons or NSCs can improve the abnormal behavior of PD model animals [6]–[9], the therapeutic effect of cell transplantation is both limited and transient due to the low survival rate of transplanted DA neurons and the low efficiency of NSCs differentiation into DA neurons in the local microenvironment of the host [10]–[14]. Thus, in order to obtain a significant therapeutic effect, cell-based therapies must not only to replace lost DA neurons but must also modify the local host microenvironment to make it more conducive to the survival and differentiation of grafted DA neurons or NSCs.

Glial-derived neurotrophic factor is the most powerful trophic factor for DA neurons [6], [7], suggesting that augmenting the local GDNF concentration may enhanced graft survival. However, GDNF alone is not easily delivered to the mesencephalon as it does not cross the blood-brain barrier [25]–[27] and has a limited half-life in vivo. Thus, alternative routes are needed [28]–[30], such as genetically modified grafted cells that stably overexpress GDNF as demonstrated in the current study.

With the advent of molecular imaging technologies, in vivo real-time monitoring of transplanted cell fate has become a reality. Cells labeled with MR contrast agents prior to transplantation can be detected by MRI [31]. In this study, we labeled neural stem cells with the superparamagnetic iron oxide (SPIO) MRI contrast agent Feridex using a liposomal transfection agent. Most mNSCs remained at the injection sites in the striatum and many expressed tyrosine hydroxylase, the rate limiting enzyme for DA synthesis. Local release of DA from these cells may account for the suppression of APO-induced rotational behavior observed in all transplantation groups, but especially in rats injected with both fetal DA neurons and mNSCs overexpressing GDNF.

In addition to a greater reduction in rotational behavior, co-transplantation of GDNF-mNSCs with fetal DA neurons improved grafted cell survival and promoted the differentiation of mNSCs into DA neurons at the transplantation site compared to transplantation of GDNF-mNSCs alone or to co-transplantation of control mNSCs with fetal DA neurons. Previous publications have reported that only 3%–20% of grafted DA neurons survived [14], and that only ≈10% of mNSCs could be induced to differentiate into DA neuron in vitro or in vivo [32], [33]. Results of our study demonstrated the survival of a significantly greater proportion of grafted cells in the GDNF-mNSCs+DA group than in the other groups. About 22.5% of GDNF-overexpressing mNSCs differentiated into DA neurons when co-grafted with fetal mesencephalic DA neurons, a rate considerably higher than in some previous reports [32]–[34]. In contrast, only 7.3% of mNSCs differentiated into (TH^+^) DA neurons when transplanted alone, more consistent with previous reports [32], [33]. Nikkhah et al. suggested that 100 surviving TH+ neurons are sufficient to produce therapeutic effects in the APO-induced rotation test [15], and we surmise that the large number of surviving TH+ neurons in our study at least partially accounts for the significant (81%) decrease in APO-induced rotational behavior in the GDNF-mNSCs+DA group compared to the sham group 8 weeks after 6-OHDA injection.

Our study also showed that some autologous NSCs (Nestin^+^/eGFP- cells) migrated to the transplantation site, which might also contribute to the recovery. We propose that the better outcome obtained by co-transplantation of GDNF-overexpressing mNSCs with fetal DA neurons is due to both GDNF expression at the transplantation site and cooperative interactions between the grafted mNSCs and DA neurons.

We demonstrate that co-injection of fetal DA neurons with mesencephalic neural stems cells overexpressing GDNF (GDNF-mNSCs) substantially enhances graft survival, NSCs differentiation to DA neurons, and functional improvement in a rat model of Parkinson’s disease. This strategy may improve the efficacy of cell-based therapies for PD.
